# Removal of Ciprofloxacin from Aqueous Solutions Using Pillared Clays

**DOI:** 10.3390/ma10121345

**Published:** 2017-11-23

**Authors:** Maria Eugenia Roca Jalil, Miria Baschini, Karim Sapag

**Affiliations:** 1 Grupo de Estudios en Materiales Adsorbentes, Instituto de Investigación y Desarrollo en Ingeniería de Procesos, Biotecnología y Energías Alternativas-CONICET, Universidad Nacional del Comahue, Buenos Aires, 1400 8300 Neuquén, Argentina; miria.baschini@fain.uncoma.edu.ar; 2 Laboratorio de Sólidos Porosos, Instituto de Física Aplicada—CONICET, Universidad Nacional de San Luis, Ejército de los Andes 950, Bloque II, 2do piso, CP 5700 San Luis, Argentina; sapag@unsl.edu.ar

**Keywords:** ciprofloxacin, adsorption, pillared clays

## Abstract

Emerging contaminants in the environment have caused enormous concern in the last few decades, and among them, antibiotics have received special attention. On the other hand, adsorption has shown to be a useful, low-cost, and eco-friendly method for the removal of this type of contaminants from water. This work is focused on the study of ciprofloxacin (CPX) removal from water by adsorption on pillared clays (PILC) under basic pH conditions, where CPX is in its anionic form (CPX^−^). Four different materials were synthetized, characterized, and studied as adsorbents of CPX (Al-, Fe-, Si-, and Zr-PILC). The highest CPX adsorption capacities of 100.6 and 122.1 mg g^−1^ were obtained for the Si- and Fe-PILC (respectively), and can be related to the porous structure of the PILCs. The suggested adsorption mechanism involves inner-sphere complexes formation as well as van der Waals interactions between CPX^−^ and the available adsorption sites on the PILC surfaces.

## 1. Introduction

The presence of antibiotics in wastewater and surface water has been widely reported [1,2,3], and is becoming a growing concern due to its toxicological effect on aquatic species as well as the resistance that they can induce on some bacterial strains even at low concentrations. These compounds reach the environment as consequence of different activities, like veterinary medicine or agriculture but, mostly, due to their use in human medicine and the inefficiency of wastewater treatments to remove this kind of contaminants, which are not biodegraded [1,2,3,4,5,6]. Consequently, different methods have been studied to eliminate antibiotics from water, such as advanced oxidation processes, nanofiltration, reverse osmosis, and photo and electrochemical degradation. The downsides of these approaches go from high maintenance costs, to complicated procedures, or secondary pollution [4,5,6].

From the available methods, the adsorption process has shown to be the most effective and inexpensive. It also involves an easier design and operation than other techniques. Moreover, there is a huge variety of adsorbents with different properties and nature, such as carbonous materials [7,8,9,10,11,12], mesoporous silica [13], hydrous oxides [14], and mineral clays [15,16,17,18,19], which have been evaluated for these new organic pollutants. Although activated carbons have been extensively used for the removal of organic compounds from water, mineral clays have acquired attention because they are effective adsorbents, as well as low-cost, widely distributed, and eco-friendly materials. In particular, bentonites have been greatly studied for removing emergent contaminants as amoxicillin [20], diclofenac potassium [21], tetracycline [22,23], cephalexin [24], and ciprofloxacin [15,17,18,19] from water. However, the bentonites bring limitations in their separation from the aqueous media due to their behavior in water suspensions. Pillared clays (PILC) are micro-mesoporous materials that are synthetized from bentonites characterized by a large specific surface area, permanent porosity, and higher hydrophobicity than the one shown by the raw material [25]. Although PILC were obtained as alternative catalysts to zeolites and have been widely studied both as catalysts and as catalyst supports, these materials have also proven to be effective adsorbents of diverse organic and inorganic pollutants [26,27,28,29,30,31,32,33,34].

Taking this into account, four pillared clays with different oligocations (Al, Fe, Si, and Zr) were synthetized and characterized. The obtained PILC were evaluated as adsorbents of ciprofloxacin from basic aqueous media in order to study the relationship between their structural and textual properties and their removal capacity.

## 2. Materials and Methods

### 2.1. Synthesis and Characterization of Pillared Clays

The natural clay (NC) used as raw material in the present work is a bentonite that is obtained from the Pellegrini lake in the province of Rio Negro, Argentina. Four pillared clays with different pillaring agents were synthetized from this NC, described in detail in a previous work [17].

The silica pillared clay (Si-PILC) was prepared following the methodology described by Han et al. [35], with some modifications. The silica sol solution that was used as pillaring agent was obtained by mixing tetraethyl orthosilicate (TEOS: Si(OEt)_4_, Merck > 99%), 2 M HCl (Cicarelli, 36.5–38%) and ethanol in a molar ratio of 1:0.1:1. The resulting solution was then aged at room temperature for 2 h and was mixed with a 0.25 M ferric nitrate (Fe(NO_3_)_3_ 9H_2_O) solution in a molar ratio of Si/Fe 10:1. This mixture was titrated with 0.2 M NaOH (Anedra, 98%) solution up to a pH of 2.7. Then, the pillaring agent obtained was added drop wise to a NC suspension of 1 wt % of deionized water in a molar ratio of Si/Fe/CIC 50:5:1, respectively. During the cation exchange, the mixture was stirred 3 h at 60 °C and the solid was separated by centrifugation at 3500 rpm for 15 min by Sorvall RC 5C centrifuge (Kendro Laboratory Product, Newtown, CT, USA). This solid (the exchanged NC), was first washed with a solution of ethanol/water 50% v/v to remove excess oligocation solution, and was then dispersed in 0.2 M HCl solution under stirring during 3 h to leach out the iron species from the mixed sol particles. This last step was carried out four more times. Finally, the solid material was washed with deionized water several times and then dried to finally obtain the Si-pillared clay precursor. 

The iron pillared clay (Fe-PILC) was synthesized according to the procedure proposed by Yamanaka et al. [36], where the trinuclear acetate-hydroxo iron (III) nitrate ([Fe_3_O(OCOCH_3_)_6_CH_3_COOH(H_2_O)_2_]NO_3_) is the pillaring agent. The latter was prepared mixing a ferric nitrate (Fe(NO_3_)_3_ 9H_2_O, Fluka, 97%) solution with ethanol and adding drop by drop acetic anhydride in a molar ratio of 1:4.3:7.4, respectively. The solution was further kept in an ice bath for cooling and the resulting precipitate was separated by filtration. Then, a 0.04 M aqueous solution of trinuclear acetate complex was prepared and added to a 1 wt % suspension of the NC in deionized water. The mixture was stirred for 3 h and the resulting suspension was then filtered, washed with deionized water, and dried, to finally obtain the Fe-pillared clay precursor. 

The aluminum pillared clay (Al-PILC) was prepared following the methodology described in previous work [32]. The pillaring agent was synthesized by basic hydrolysis of AlCl_3_ · 6H_2_O (Anedra, Lieshout, NL, 99.5%) solution with NaOH solution. The basicity relationship was of OH^−^/Al^3+^ = 2 and the mixture was initially maintained under stirring at 60 °C, and then aged under stirring for 12 h at room temperature. The pillaring agent obtained was added drop wise to a 3 wt % dispersion of NC in deionized water and it was stirred for 1 h. The resulting solid was washed using dialysis membranes and was dried to obtain the Al-PILC precursor. 

The zirconium pillared clay (Zr-PILC) was synthesized using the methodology proposed by Farfan Torres et al. [37] with modifications. The pillaring agent used was a ZrOCl_2_ 8H_2_O (Merck, Kenilworth, NJ, USA, 99.9%) solution initially adjusted at a pH of 1.9 using a NaOH solution. The resulting solution was added drop by drop to a 1 wt % suspension of NC in deionized water under stirring at 40 °C. The mixture was then stirred for 2 h, filtered, washed with deionized water, and dried for obtaining the Zr-PILC precursor.

Finally, all of the precursors of the PILC were calcined at 500 °C for 1 h in order to obtain the Si-PILC, Fe-PILC, Al-PILC, and Zr-PILC samples.

The structural properties for all of the materials were analyzed by X-ray Diffraction (XRD) using a RIGAKU Geigerflex X-ray diffractometer (Rigaku, Austin, TX, USA with CuKα radiation at 20 mA and 40 kV. The scans were recorded between 2° and 70° (2θ), with a step size of 0.02° and a scanning speed of 2° min^−1^. The textural properties were studied by nitrogen adsorption-desorption isotherms at −196 °C. These measurements were carried out using an Autosorb 1 MP and iQ (Quantachrome, Boynton Beach, FL, USA). All of the samples were previously degassed for 12 h up to a residual pressure lower than 0.5 Pa at 200 °C. Textural properties were obtained from these isotherms by different methods. The specific surface area (S_BET_) was assessed by the Brunauer, Emmet and Teller (BET) method, using the Rouquerol’s criteria [38]. The micropore volumes (V_μp_) were calculated with the *α-plot* method using the corresponding sample calcined at 1000 °C as reference material [39]. The total pore volume (V_T_) was obtained using the Gurvich rule (at 0.97 of relative pressure) [38]. Pore size distributions (PSD) were obtained by the Horvarth-Kawazoe method, when considering the adsorption branch and that the PILC have slit shape pores within the interlayer region.

### 2.2. Adsorptive: Ciprofloxacin

**Figure 1:**
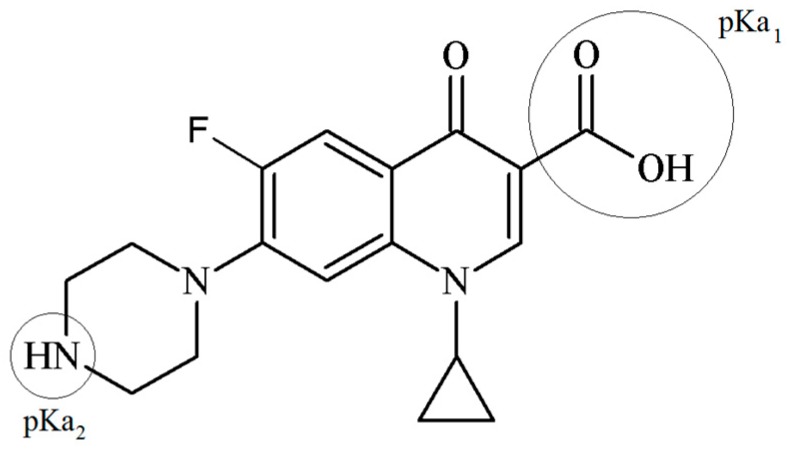
Ciprofloxacin structure.

The ciprofloxacin (CPX) is an antibiotic from the fluoroquinolones group, which is widely used in human health. The CPX is a crystalline solid with a molecular weight of 331.4 g mol^−1^. The dimensions of the (CPX) molecule are 1.35 nm × 0.3 nm × 0.74 nm and a scheme of its chemical structure is shown in Figure 1 [8].

The presence of protonable groups in the CPX structure generates two pKa values, and, in consequence, three possible species in solution. The pKa values for the CPX are 5.90 ± 0.15 (pKa_1_) and 8.89 ± 0.11 (pKa_2_), which correspond to the carboxylic acid group and the amine group in the piperazine moiety, respectively (see Figure 1). The protonation-deprotonation reactions that take place at different pH values of the media affect the species that are present in the solution, as well as their solubility. Therefore, the CPX can be found as cation (CPX^+^), zwiterion (CPX^±^), or anion (CPX^−^) under different pH values where the zwiterion form shows the lowest solubility [17].

In this work, the ciprofloxacin hydrochloride was used as adsorptive; it was acquired from Romikim S.A (CHEMO Argentina, Buenos Aires, Argentine) and had 99.3% of purity.

### 2.3. CPX Adsorption Studies

The adsorption experiments were conducted by mixing 0.02 g of adsorbent with 8 mL of CPX solutions from 18 to 230 mg·L^−1^ in tubes of 10 mL and further stirring at 20 °C up to beyond the equilibrium time. The values were chosen according to previous results [17]. In all of the tests, the tubes were wrapped in aluminum foils to prevent light-induced decomposition. After the adsorption, the solutions were separated from the adsorbent using a Sorvall RC 5C centrifuge at 8000 rpm for 20 min. The CPX equilibrium concentrations in the resultant supernatant were measured with a T60 UV-vis spectrophotometer (PG Instruments Lmited, Leicester, UK) at the λ_max_ corresponding to the pH value, from the previously determined calibration curve. The absorption spectra were obtained for every point of each isotherm. In all of cases the spectra observed were the same as those of CPX before clay contact, evidencing that CPX is not degraded after contact with PILC materials. All of the samples were measured in duplicate and the average value was used. The amount of CPX adsorbed on the clay mineral (*q*) was calculated from the initial and equilibrium CPX concentrations, according to an Equation (1):

(1) $$q=\frac{V\left(C_{i}-C_{eq}\right)}{w}$$

where *V* is the CPX solution volume (L), *C_i_* is the initial CPX concentration (mg L^−1^), *C_eq_* is the equilibrium CPX concentration (mg L^−1^), and *w* is the mass of clay (g).

### 2.4. Modelling Methods

Langmuir, Freundlich, and Sips isotherms models were fitted to the CPX adsorption equilibrium data [40]. The Langmuir model assumes a monolayer adsorption on a surface with a finite number of identical sites, which are energetically equivalent and where there are no interactions among the adsorbed molecules (homogeneous surface). The mathematical expression of the Langmuir model is shown in Equation (2):

(2) $$q=\frac{q_{m}{kC}_{eq}}{{1+kC}_{eq}}$$

where *q_m_* is the maximum adsorbed concentration within a monolayer of adsorbate (mg g^−1^) and *k* (L mg^−1^) is the Langmuir adsorption equilibrium constant, which is related to the adsorption energy.

The Freundlich equation is an empirical method that has been widely applied to adsorption on heterogeneous surfaces. This model uses a multi-site adsorption isotherm and its mathematical expression is defined in Equation (3).

(3) $${q=k}_{F}C_{eq}^{1/n}$$

where *k_F_* (mg g^−1^(L mg^−1^)^n^) and *n* (dimensionless) are the Freundlich characteristic constants, indicating the adsorption capacity and adsorption intensity, respectively.

The Sips equation is a combination of the Langmuir and Freundlich equations. It is an empirical equation that assumes a heterogeneous surface with a number of active sites that interact with the adsorbate molecule, without adsorbate-adsorbate interactions. The mathematical expression is shown in Equation (4).

(4) $${q=q}_{m}\frac{{{(bC}_{eq})}^{1/n}}{{1+(bC}_{eq}{)}^{1/n}}$$

where *q_m_* and *C_eq_* have the same meanings as above, *b* is a parameter related to the affinity of the adsorbate towards the surface, and *n* is a parameter that represents the heterogeneity of the system.

The Scatchard model was applied to the adsorption data to gather complementary information about the adsorption phenomena. This method involves the transformation of the data from the isotherm to obtain a plot of *q*/*C_eq_* versus *q* (where *q* has the same meaning indicated above). The resulting plot is called a Scatchard plot and if it is a straight line, it suggests that the adsorption takes place in the same type of sites. On the other hand, if the plot is a non-linear curve, its shape can be related to nonspecific or multi-type interactions between the adsorbate and adsorbent surface. Concave curves are related to negative cooperative phenomena or to the presence of heterogeneity sites for the adsorption. In turn, convex curves are associated to positive cooperative phenomena where the first adsorption occurs with low affinity and that the adsorbate becomes a possible site for the subsequent adsorption. Additionally, any deviation from linearity in the Scatchard plot (taking R^2^ values) could be considered as an indication of the presence of nonspecific or multi-type interactions of the adsorbate molecules towards the surface sites [41,42,43,44].

## 3. Results and Discussion

### 3.1. Adsorbents Characterization

XRD patterns obtained for the PILC materials were evaluated in contrast to those obtained for the NC and the basal distances (*d*_001_). The *d*_001_ value obtained for NC was 1.26 nm, which is typical of natural sodic montmorillonites, whereas Al- and Fe-PILC showed values of 1.85 and 1.60 nm, respectively. These increases in the basal distances for the PILC could be attributed to the presence of aluminum and ferric oxide species within the NC interlayer. Additionally, the diffractograms did not show any other structural changes in the PILC in relation to the starting material. In the case of the Si- and Zr-PILC, the patterns obtained do not show any defined peaks. The low resolution for these materials could be due to a higher heterogeneity in their structures in comparison to the NC. This heterogeneity was possibly caused by a stronger acid media used in their synthesis, which may have affected the usual order of the raw material layers [45,46].

**Figure 2:**
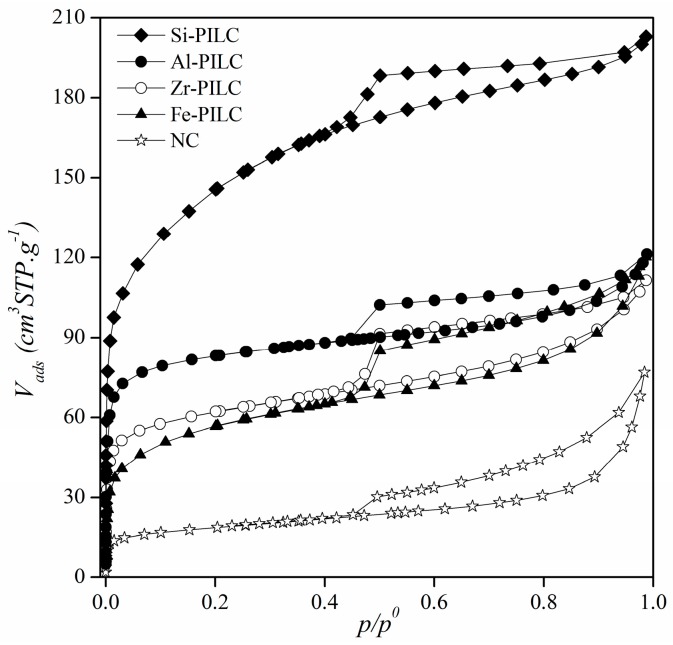
N_2_ adsorption-desorption isotherms at 77 K for natural and pillared clay minerals.

Nitrogen adsorption-desorption isotherms at −196 °C of the adsorbents are shown in Figure 2, where the amount adsorbed is expressed in cubic centimeters at STP (standard temperature and pressure) per gram of material (V_ads_ cm^3^, STP g^−1^) plotted against the equilibrium relative pressure (*p*/*p*^0^). The shape of adsorption-desorption isotherms are grouped into six types and can be related to particular pore structures according to the IUPAC (International Union of Pure and Applied Chemistry) classification [38,47]. Taking this in account, the natural clay isotherm can be classified as type IIb isotherm with an H3 hysteresis loop. This kind of isotherm is associated to mesoporous materials with aggregates of plate-like particles, like the montmorillonites. The isotherms that are obtained for the pillared clays can be classified as a combination between type I, at low relative pressures, and type IIb due to the adsorption behavior in the mono-multilayer region (from 0.05 to ca. 0.8 in relative pressures). The high amount adsorbed at low relative pressures is related to the microporosity that is generated as a result of the pillaring process. In addition, the presence of mesoporosity in PILC is evidenced by the presence of the type H4 hysteresis loops that are associated to slit-like pores, as well as to the pores that are generated within the interlayer of the clay minerals. Among the pillared clays, the Si-PILC showed the highest adsorption values at low relative pressure, suggesting that this material developed a greater microporosity. Regarding Al-, Fe-, and Zr-PILC, the adsorption in the micropores region was lower than the one that was obtained for the Si-PILC, but was still higher than the one obtained for the natural clay, suggesting a successful pillaring process.

The textural properties obtained for all of the materials from nitrogen adsorption-desorption isotherms are summarized in Table 1.

Data showed an increase in the specific surface areas (S_BET_) for all PILC in comparison with the raw material, and, due to the microporosity accomplished by the pillaring process. The highest and lowest S_BET_ values were eight and three times the NC value for Si- and Fe-PILC, respectively. The order of increase of S_BET_ values was the same as the micropores volume (V_μp_) for all of the materials, where the values obtained for Si- and Al-PILC were around two times higher than the ones that were obtained for other PILC. This suggests a higher density of pillars for these materials than for the other PILC, and it could be related to the synthesis method. The percentages of microporosity were between 41% and 66% for Zr- and Al-PILC, respectively. Si- and Al-PILC were the materials exhibiting the highest microporosity percentages, suggesting pillaring agents with more uniform structures and greater pillars density within the interlayer. The V_T_ values supported the behavior of the isotherms at high relative pressures. The highest value was obtained for the Si-PILC, suggesting a higher porosity in this sample as compared to the other PILC.

**Figure 3:**
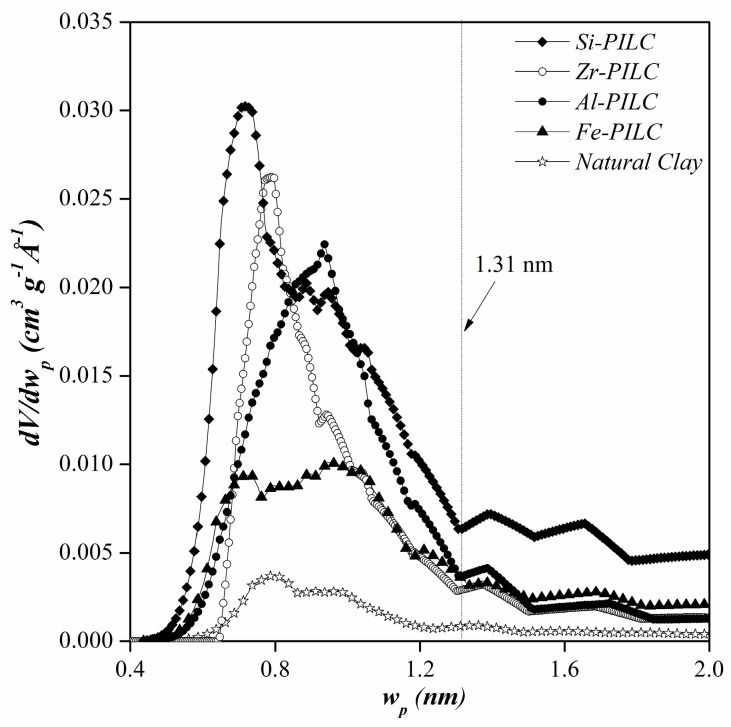
Pore side distribution of natural and pillared clay minerals where *V* is adsorbed volume and *w_p_* is the pore size.

The pore size distributions (PSD) for the PILC are shown in Figure 3. All of them were studied in the microporous region (pores size below 2 nm) to comparatively follow the development of microporosity. Al-PILC has micropore sizes of between 0.4 and 2 nm and are higher than the one observed for the NC. However, Si- and Zr-PILC showed PSD with more defined micropore sizes around 0.6 and 0.8 nm, respectively. These results could suggest that a high density of pillars was caused during the pillaring process in these materials. The PSD obtained for the Al-PILC shows that this material has micropore sizes of around 0.9 nm, which are the characteristic dimensions of the kegging cation that is used as pillaring agent [32]. The Fe-PILC showed fewer micropores than the other PILC and the PSD shape suggested micropores with different sizes in its interlayer. All of the results showed an agreement with the textural properties.

### 3.2. Effect of pH Media on Adsorption

**Figure 4:**
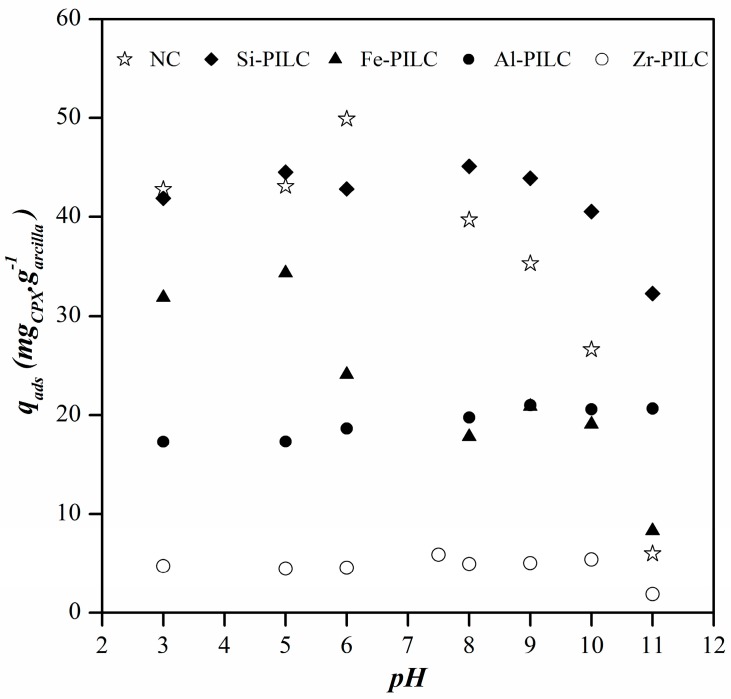
Effect of media pH on ciprofloxacin (CPX) adsorption.

The studies of CPX adsorption on NC and PILC at different pH values were assessed under the above mentioned conditions, with an initial fixed CPX concentration of 110 mg L^−1^. The pH of the initial solution was adjusted to values between 3 and 12 using HCl or NaOH solutions. This value was chosen based on the CPX solubility that was measured in previous work [17]. Adsorption capacities of CPX on NC and PILC at different pH values are shown in Figure 4.

The results obtained for NC exhibited highest CPX adsorption at low pH values, decreasing with the pH increase from 7.5, which can be explained by the relationship between the surface charge of NC and the CPX species that are present. At low pH values, the species of CPX that are present are in their cationic form, favoring the adsorption on negative charged NC surface by cation exchange of CPX^+^ for the natural cation within the montmorillonite interlayer. This is the adsorption mechanism that is proposed and widely reported for adsorption of cations on montmorillonites [17,18,22,48]. The decrease in CPX adsorption after a pH of 7.5 can be explained by the presence of the zwiterionic and anionic forms of CPX. In these cases, the presence of a negative charge in the CPX structure results in repulsive interactions with the mineral negative surface, resulting in other adsorption mechanisms. The highest amount adsorbed for the NC was obtained at a pH of 6, probably due to the competitive adsorption of the H^+^ against the CPX^+^ species towards the same sites on the clay surface [17].

The results for the pillared clays showed different behaviors according to the pillaring agent. The Fe-PILC exhibited the highest amount adsorbed at low pH, abruptly decreasing as the pH increases. This behavior can be explained by the interaction between the negative surface of the Fe-PILC and the positive species of CPX, favoring the adsorption at pH values lower than 5 [49]. Similar adsorption results were reported for rhodamine B and diclofenac on Fe-PILC [21,27]. For the Si-PILC, results showed no significant variations in the amount adsorbed at pH values below 8, and decreasing as the pH increases. However, the amounts adsorbed on the Si-PILC were much higher if compared to the adsorption of the other materials in the alkaline media. These results could suggest that Si-PILC has more available surface sites than the other PILC in this pH range, favoring the adsorption of CPX anionic species. On the other hand, the Si-PILC was the material with the highest micropores amount and narrow microporosity, both could be responsible for the increase in adsorption. These results are pretty interesting since there are no reports of Si-PILC being studied as adsorbents. The adsorption behaviors for Al- and Zr-PILC were similar, with no major differences in the amount adsorbed across the pH range. This behavior could suggest that for these types of materials, the adsorption mechanism is mainly governed by their porous structures limiting the access of the CPX molecule to the pillared structure. Analogous results were reported by Gil et al. [26] for the adsorption of orange II and methylene blue on Al- and Zr-PILC. That study took into account that the adsorption of a molecule occurs in pores with a diameter 1.3–1.8 times that of the solute. If this criterion is taken into account, and, since the adsorptive will diffuse into the porous structure of the adsorbent lengthwise, the minimum pore size for the adsorption of the CPX should be 1.31 nm. If that is the case, the adsorption results could be explained by Figure 3, where the aluminum and zirconium pillared clays are the materials with the lowest amount of pores that are higher than this size, similar to Fe-PILC, whereas the Si-PILC has higher amounts of pores in this range. This may indicate that the CPX molecule has more access to the pillars in the last material, which, in turn, could favor the interaction and adsorption between them.

The results obtained for the CPX adsorption on the PILC at different pH values suggest that some pillared materials would be optimal adsorbents of the CPX anionic specie. With that in mind and considering that the pH value of the natural water courses in the Alto Valle region is around 9, the adsorption and kinetics studies were carried out at pH of 10.

### 3.3. Adsorption Isotherms

The batch adsorption experiments were performed in the conditions previously mentioned, varying the initial concentrations between 18–500 mg L^−1^ and the contact time, now set for a period of 24 h. The adsorption studies were ran at pH 10, based on the results for CPX adsorption at different pH values and in order to evaluate the behavior of the pillared clays when the CPX anionic form is present. According to previous kinetic studies (not shown in this work), the optimal contact time was 24 h.

**Figure 5:**
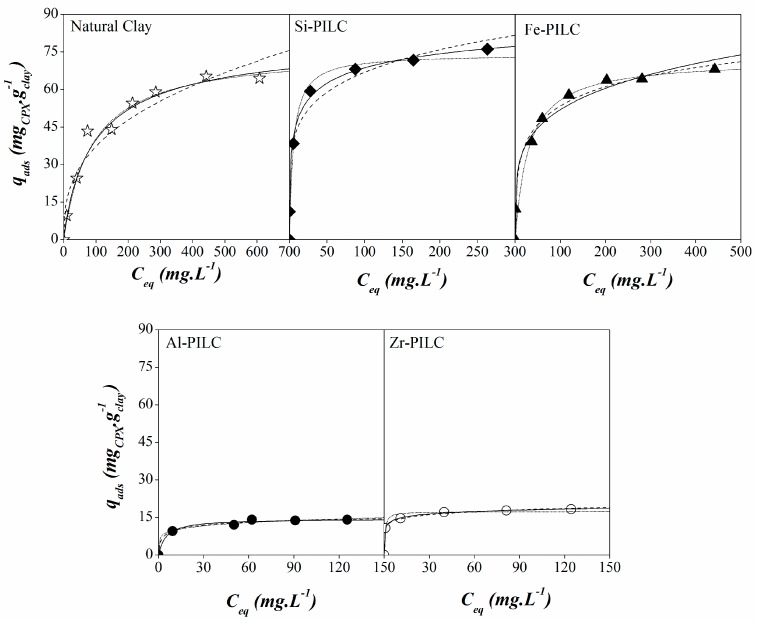
Experimental isotherms (symbols) and Langmuir (dash), Freundlich (dot) and Sips (straight) adjustments for the equilibrium adsorption data of CPX on natural clay (NC) and pillared clays (PILC).

The adsorption isotherms obtained for CPX on different clays and their adjustments to the three models, are shown in Figure 5. Taking into account the Giles et al. classification [50], two different behaviors can be associated to the isotherm shapes illustrated there. The adsorption isotherms for CPX on pillared clays can be classified as high affinity type (H-type) and the one obtained for the natural clay was a Langmuir type (L-type) isotherm. In both of the cases, the isotherm shape is related to a progressive saturation of the solid surface due to the occupancy of the adsorbent surface sites, suggesting a high affinity of the adsorptive molecule toward the solid surface. The H-type isotherm is usually associated to the ionic solute adsorption where there is no strong competition between adsorptive and solvent molecules towards the surface of the solid [50,51]. This could be the result of a higher hydrophobicity being exhibited by the pillared clays in contrast with the natural clay. Another explanation is the presence of new adsorption sites in the pillared clays surface associated to the pillars. On the other hand, the L-type isotherm for the natural clay suggests a lower affinity of the anionic CPX species present toward the more negatively charged clay than the one that is observed for the PILC.

Freundlich, Langmuir, and Sips models were fitted to adsorption data obtained for all of the materials, and their fitting parameters are summarized in Table 2. The best fittings were obtained for the Sips model in all of the materials that were under study. This suggests heterogeneous systems that could result from the presence of different adsorption sites on the solid surface, the adsorbible species, or a combination of them. Similarly, when the parameter associated to the system heterogeneity in this model (*n*) is 1, the Sips equation becomes the Langmuir equation and the system can be considered to be a more homogeneous one. Thus, the *n* value nearest to 1 obtained for the natural clay suggests an adsorption system that is more homogeneous when compared to the values obtained for the PILC, which are greater than 2 in all of the cases. This indicates a more heterogeneous system, which is probably due to the PILC porous structure and the pillars presence. The highest adsorption capacity was obtained for Si-PILC and the lowest for Zr-PILC. The natural clay resulted in an intermediate adsorption capacity between two groups of pillared materials, Si- and Fe-PILC, which showed higher adsorption capacities and Al- and Zr-PILC, which were significantly lower. However, the affinity of the adsorbate for the solid surface is lower for the NC than it is for the pillared clays. Based on these results, the adsorption capacity for the NC could be explained by a hydrophobic effect of the solvent towards the organic molecule where the adsorption could be seen as a result of the repulsion of the organic molecule against the solvent from the solution [52]. The hydrophobic effect can be related to the k_ow_ value for the CPX, which is 1.9, indicating the hydrophobic character of the CPX molecule. After the molecule is in the solid-liquid interface, different adsorption short-range forces could be promoted between the CPX molecule and the solid surface, such as covalent and hydrophobic bonding, hydrogen bridges, steric, or orientation effects [52]. Additionally, the type L isotherm that is obtained is associated to a flatwise adsorption favoring van der Waals (π–π type) interactions between the aromatic fraction in the organic molecule and the siloxane surface of the clay material [48,50]. In the pillared clays, the hydrophobic effect could influence the adsorption the same way that it did for the NC. However, there are two additional factors that affect the adsorption capacity in these materials; the new adsorption sites that are generated by the pillars presence and the porous structure associated to them. The adsorption behaviors obtained are consistent with the results that are shown for the adsorption vs. pH, meaning that the highest adsorption capacity was obtained for the Si-PILC, whereas the lowest one was for Al- and Zr-PILC. The adsorption shown for the Si- and Fe-PILC could be due to a higher access of the CPX species to the porous structure when compared to the other PILC favoring its interaction with the different adsorption sites within the interlaminar region.

**Figure 6:**
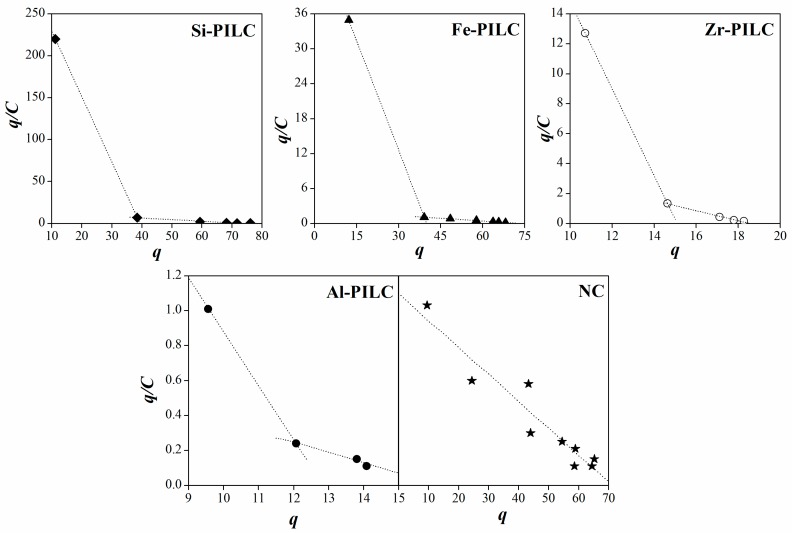
Scatchard plots derived for adsorption data obtained at pH 10 for the five materials.

The Scatchard plots obtained for all of the materials are shown in Figure 6. The R^2^ values that are obtained for the whole range of data could suggest the presence of nonspecific or multi-type interactions between the adsorbate molecules and the surface sites. The R^2^ values calculated were 0.906, 0.887, 0.856, 0.753 and 0.739 for AN, Al-PILC, Zr-PILC, Fe-PILC and Si-PILC, respectively. These values indicate a greater presence of nonspecific interactions for all PILC materials than there are for the NC, being the highest, the ones obtained for Fe- and Si-PILC. Furthermore, the Scatchard plots obtained for all the pillared clays can be considered as concave curves that are associated to a negative cooperative adsorption phenomenon, as well as surface heterogeneity [42,43]. As it can be seen in Figure 6, the Scatchard plots obtained for the PILC materials result in two independent sets of data, which individually arrange in a linear combination, where each one of them could be related to a type of affinity of the CPX specie to the surface. This may be the result of the presence of different adsorption sites in the clay surface causing the CPX^−^ to show high (H) and low (L) affinities towards the PILC surface [41,43]. These results could suggest that at an early stage of the adsorption process, the CPX^−^ interacts with the pillars either through the non-bonding electrons in its amine group or through the electrons of its carboxylate group, both with high affinity. However, this access is limited to a small amount of sites that become quickly saturated. Afterwards, the CPX species are adsorbed on the available sites of the clay surface by other types of low affinity interactions (i.e., van der Waals (π–π type) interactions, hydrogen bridges, etc.). On the other hand, the Scatchard plot that is obtained for the natural clay is a straight line, which is associated to an adsorption process where the solid surface only exhibits one type of site for the CPX anion to be adsorbed.

The results show a close relationship between the adsorption capacity of the PILC materials and their porous structure, as well as the influence of the micropores size on the CPX^−^ access to the interlaminar space for its subsequent adsorption. However, in order to obtain complementary information about the possible contribution of the mesopores present in the materials structure on the CPX^−^ adsorption, textural properties were evaluated. Table 3 shows the values of the cumulative volumes for the pillared clays when considering three ranges; (1) the amount of micropores whose size is higher than 2 nm (V_µp_ < 2 nm), (2) the mesopores ranging between 2–10 nm, and the (3) the mesopores between 10 and 50 nm. The values show that the materials with the highest adsorption capacities (Si- and Fe-PILC) also have the highest amount of mesopores with a size of lower than 10 nm. This indicates that the mesopores are playing an important role in the CPX adsorption on pillared clays under the studied conditions. This type of pores might be more accessible for the molecule and represent adsorption sites for the kind of interactions mentioned earlier.

Table 4 summarizes the results reported for other authors for the adsorption of CPX on different adsorbents, and they are compared to the results obtained in this work. It shows that most of the reported studies were carried out at pH values lower than 7 and under these conditions the materials with highest adsorption capacity of CPX are the clay minerals. These results probably are because at those pH values the cationic species of CPX is present and it has high affinity for the negative clay minerals surfaces. However, there are less reports of the CPX adsorption at pH values higher than 7 when the anionic species is present. In this sense, the adsorption capacity of the natural clay mineral was lower than the results showed for the pillared clays, suggesting that these materials could be good adsorbents for anionic species.

### 3.4. Evidences of CPX Interactions with PILC

Looking for evidences of the interactions between the CPX species and the pillared clays surface, FTIR (Fourier-transform infrared spectroscopy) spectra of adsorbed CPX on the PILC (adsorption complex) were obtained. The adsorption complexes were studied for the saturated points in the adsorption isotherms, after the centrifugation step, each one of them was dried at room temperature and the FTIR of the resultant solids were obtained. The spectra were compared with those obtained for the CPX (pure) and for the PILC materials.

**Figure 7:**
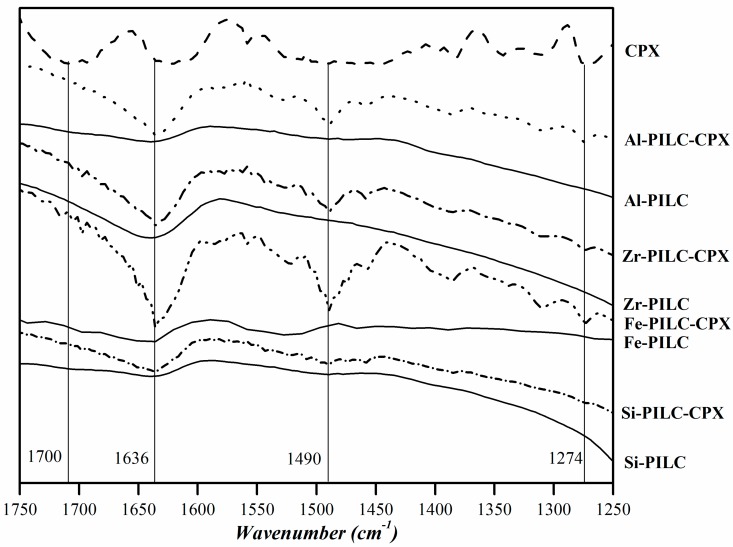
FTIR (Fourier Transform infrared spectroscopy) spectra of CPX, PILC, and the adsorption complexes obtained.

**Figure 8:**
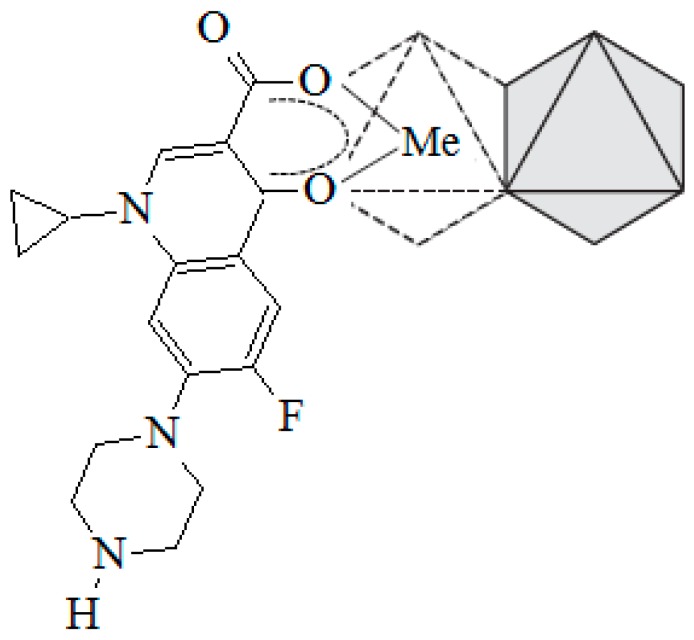
Representation of the structure proposed for the interaction between CPX^−^ and atoms of metals on the PILC surface.

Figure 7 shows the resultant spectra for all of the samples CPX, PILC materials, and adsorption complexes. The vibration bands that were obtained for the pure CPX are comparable with the ones that were obtained in the previous work [17]. In the same sense, the vibration bands that are associated to the interaction between the CPX species and different solids and metals have been previously reported [14,54]. Taking these works into account, the bands at 1264 and 1700 cm^−1^ found in the CPX spectrum are assigned to the protonation of the carboxylic group and the stretching of its carbonyl group, respectively. After the adsorption, the first band shifted to 1274 cm^−1^ for all of the adsorption complexes, suggesting that the carboxylate group is involved in the adsorption process. This possibly occurs by Lewis acid-base interactions between its electrons and the metallic atoms that are available in the solid surface. The second band is missing, whereas two bands appear at 1636 and 1490 cm^−1^ for the adsorption complexes that are assigned to the asymmetric and the symmetric stretch of the coordinated carboxylate group of the CPX molecule. The presence of these last bands could be associated to the CPX^−^ acting as a mononuclear bidentate ligand in the adsorption complexes where the oxygen of the carbonyl group, belonging to the quinolone moiety, and one of the oxygen atoms of the carboxylate group interact with the available metallic atoms on the pillared clays surface. Those kinds of complexes have been proposed previously by other authors for the adsorption of CPX on aluminum and iron hydrous oxides [14]. In view of this evidence, and considering the previous works, the possible structure for the interaction between CPX and the metal atoms on the solid surface is represented in Figure 8.

## 4. Conclusions

In this study, different pillared clay minerals were evaluated as adsorbents of ciprofloxacin in basic conditions. The highest CPX adsorption capacity was obtained for the Si- and Fe-PILC at the studied conditions and it could be related to both, the presence of micro and mesoporous with sizes greater than the Al- and Zr-PILC, and the new adsorption sites that are generated for the metal atom in the pillars. Adsorption data evidenced that there is a strong relationship between the porous structure of the pillared clay and their adsorption capacity, suggesting that the latter could be mainly related to the access of the CPX molecule to the pillars within the PILC interlaminar region. Additionally, the results suggest that the adsorption mechanism for the CPX on the PILC involves a first moment governed for the hydrophobic effect on CPX^−^ followed by the adsorption that is caused by the inner-sphere complexes formation, as well as by the van der Waals interactions between CPX^−^ and the PILC sites surface.

## Figures and Tables

**Table 1:** Textural properties data and d_001_ obtained for natural and pillared clays.

Materials	S_BET_ (m^2^ g^−1^)	V_T_ (cm^3^ g^−1^)	V_µp_ (cm^3^ g^−1^)
Natural Clay	67	0.10	0.01
Al-PILC	322	0.18	0.12
Si-PILC	519	0.31	0.19
Zr-PILC	231	0.16	0.07
Fe-PILC	206	0.17	0.07

**Table 2:** Freundlich, Langmuir and Sips parameters for CPX adsorption on natural and pillared clay minerals.

Models	Units	NC	Si-PILC	Fe-PILC	Al-PILC	Zr-PILC
Freundlich model	*k_F_* (mg g^−1^(L mg^−1^)^*n*^)	6.98	28.88	18.98	6.86	11.22
	n	2.75	5.49	4.57	6.45	9.48
	R^2^	0.962	0.979	0.986	0.986	0.997
Langmuir model	*q_m_* (mg g^−1^)	75.73	74.12	72.09	14.48	17.34
	*K* (L mg ^−1^)	0.01	0.19	0.03	0.20	1.78
	R^2^	0.993	0.971	0.966	0.986	0.975
Sips model	*q_m_* (mg g^−1^)	80.82	100.60	122.10	17.78	25.20
	*b* (L mg ^−1^)	0.01	0.07	0.01	0.14	0.36
	*n*	1.13	2.56	2.76	2.07	3.85
	R^2^	0.993	0.996	0.991	0.987	0.999

**Table 3:** Cumulative volumes for the pillared clays.

Pillared Clays	V_µp_ (<2 nm)	V_mp_ (2–10 nm)	V_mp_ (10–50 nm)	V_T_
Si-PILC	0.09	0.18	0.04	0.31
Fe-PILC	0.03	0.08	0.06	0.17
Al-PILC	0.10	0.04	0.04	0.18
Zr-PILC	0.06	0.05	0.06	0.17

**Table 4:** CPX adsorption capacities for different materials.

Adsorbent	*q_m,CPX_* (pH) (mg g^−1^)	Reference
Aluminum hydrous oxide	14.72 (7)	[14]
Iron hydrous oxide	25.76 (7)	
Ca^2+^-montmorillonite (Saz)	330 (4–5.5)	[19]
Activated carbon	231 (≈7)	[8]
Carbon nanotubes	135 (≈7)	
Carbon xerogel	112 (≈7)	
kaolinite	6.99 (5–6)	[53]
Illite	33 (4–5.5)	[18]
Rectorie	135 (4–5.5)	
Bentonite	147 (4.5)	[15]
Birnessite	80.96 (5–6)	[16]
Montmorillonite	332.8 (3) 138.7 (6) 71.6 (7.5) 80.82 (10)	[17]
Graphene Oxide	379 (5)	[11]
CMK-3 CMK-3 modified Bamboo-based carbon Bamboo-based carbon modified	281.47 (<7) 369.34 (<7) 153.17 (<7) 237.44 (<7)	[10]
Multi-walled nanotubes	194 (4)	[12]
Si-PILC	100.6 (10)	This work
Fe-PILC	122.1 (10)	This work
Al-PILC	17.78 (10)	This work
Zr-PILC	25.20 (10)	This work
